# Postoperative drainage with double 8F ultrafine chest tubes improves pain control and reduces specific complications in uniportal thoracoscopic lung tumor resection: a retrospective multicenter cohort study

**DOI:** 10.3389/fmed.2026.1806379

**Published:** 2026-04-24

**Authors:** Hongde Jiang, Di Wu, Zhengwei Huang, Donghui Chu, Jun Wang, Zufang Liao, Liwei Tang, Wentao Hu, Guofang Zhao, Zhengfu He, Chenwei Li

**Affiliations:** 1 Health Science Center, Ningbo University, Ningbo, China; 2 Department of Thoracic Surgery, The First Affiliated Hospital of Ningbo University, Ningbo, China; 3 Department of Thoracic Surgery, Sir Run Run Shaw Hospital, Zhejiang University, Hangzhou, China; 4 Department of Thoracic Surgery, Ningbo Second Hospital, Ningbo, China

**Keywords:** U-VATS, lung tumor, ultrafine chest tube, postoperative drainage, perioperative outcomes, 1:1:1 PSM

## Abstract

**Background:**

Uniportal video-assisted thoracoscopic surgery (U-VATS) is a well-established minimally invasive approach for lung tumors, but consensus on the optimal size and number of postoperative chest tubes remains lacking. Ultrafine pigtail chest tubes may reduce tissue injury and improve wound healing compared with traditional drainage methods, yet evidence supporting their safety and feasibility in U-VATS patients is insufficient.

**Methods:**

This retrospective multicenter cohort study enrolled 1,076 lung tumor patients who underwent U-VATS across three Chinese hospitals. Patients were assigned to three groups: double 8F ultrafine chest tube (*n* = 427), 22F + 8F chest tube (*n* = 452), and single 24F chest tube (*n* = 197). Perioperative outcomes were analyzed using 1:1:1 propensity score matching (PSM) and linear regression models to adjust for confounders.

**Results:**

Multivariate analysis identified chest tube characteristics, pleural adhesions, postoperative infection, air leakage, intrathoracic hemorrhage, drainage volume as independent factors associated with drainage duration. After PSM (93 cases/group), the double 8F group had significantly lower NRS pain scores [postoperative days (POD) 1–3], reduced early drainage volume (POD1 and POD3), and lower incidences of atelectasis and intrathoracic hemorrhage (all *p* < 0.05) compared with the 22F + 8F group. It showed comparable hospital stay and total drainage volume to the other two groups. The single 24F group had the shortest drainage duration (*p* < 0.001), with no intergroup differences in infection, air leakage, reintubation, or chylothorax.

**Conclusion:**

Double 8F ultrafine chest tubes do not shorten drainage duration but effectively alleviate postoperative pain (especially on POD1), reduce early postoperative drainage volume, and lower specific complications. Aligning with enhanced recovery after surgery (ERAS) principles, they represent a promising drainage strategy for lung tumor patients after U-VATS.

## Introduction

U-VATS has become a well-established minimally invasive technique for lung tumor resection, offering advantages of reduced postoperative pain, shorter drainage duration and hospital stay over thoracotomy and multi-port VATS (1–4), and aligning with ERAS principles. Thoracic drainage tube insertion is an essential postoperative procedure for U-VATS, serving to remove intrathoracic blood, gas and exudate, restore pleural negative pressure, promote lung re-expansion and prevent infection (5, 6). However, there remains no clinical consensus on the optimal size, number and type of chest tubes for U-VATS, with clinical selection often relying on surgeon preference (7, 8), hindering standardized perioperative management.

Chest drainage tubes in thoracic surgery are categorized by caliber and structure into large-bore tubes (≥20F, e.g., 22F, 24F), small-bore tubes (10F ~ 20F) and ultrafine pigtail catheters (≤8F). Large-bore tubes are the traditional choice, providing efficient drainage of high-volume/viscous effusions but causing severe intercostal nerve compression and tissue irritation, which leads to intense postoperative pain, restricts respiratory exercises and delays recovery; their larger insertion incisions also impede wound healing. Small-bore tubes balance trauma and drainage efficacy but offer limited pain relief, while ultrafine 8F pigtail catheters feature a soft texture and pigtail distal design (preventing displacement/kinking), minimizing thoracic tissue injury and postoperative pain, and facilitating early functional exercises. However, clinical concerns persist about whether their ultra-fine caliber ensures sufficient drainage and avoids blockage.

Tube caliber (8F, 22F, 24F) directly impacts drainage efficiency, postoperative pain and recovery. 24F and 22F large-bore tubes (Supplementary Figure S1) yield optimal drainage but the worst pain; 8F ultrafine tubes (Supplementary Figure S1) excel in pain relief but their drainage efficacy and safety in U-VATS remain unconfirmed. While pigtail catheters have been shown to reduce wound injury compared with traditional drainage (9), existing evidence for ultrafine 8F catheters in U-VATS is limited to small single-center studies, with no rigorous multicenter research on the novel double 8F ultrafine chest tube strategy—which may combine ultrafine caliber pain relief and dual-tube drainage efficiency.

Critical knowledge gaps persist in the current literature regarding double 8F ultrafine chest tubes: whether they can achieve drainage efficacy non-inferior to conventional approaches while mitigating postoperative pain; their specific effects on early postoperative drainage volume and distinct postoperative complications; and whether this drainage strategy aligns with ERAS principles and can be standardized for U-VATS.

This multicenter observational study thus evaluated the efficacy and safety of double 8F ultrafine chest tubes for postoperative drainage after U-VATS (lobectomy, segmentectomy, wedge resection) for lung tumors, and compared perioperative outcomes with 22F + 8F combined tubes and single 24F large-bore tubes. We identified independent predictors of drainage duration and clarified the clinical advantages of double 8F tubes in pain control and complication reduction, aiming to fill the above knowledge gaps and provide high-quality clinical evidence for standardized chest tube selection in U-VATS.

## Methods

### Patients

**Figure 1:**
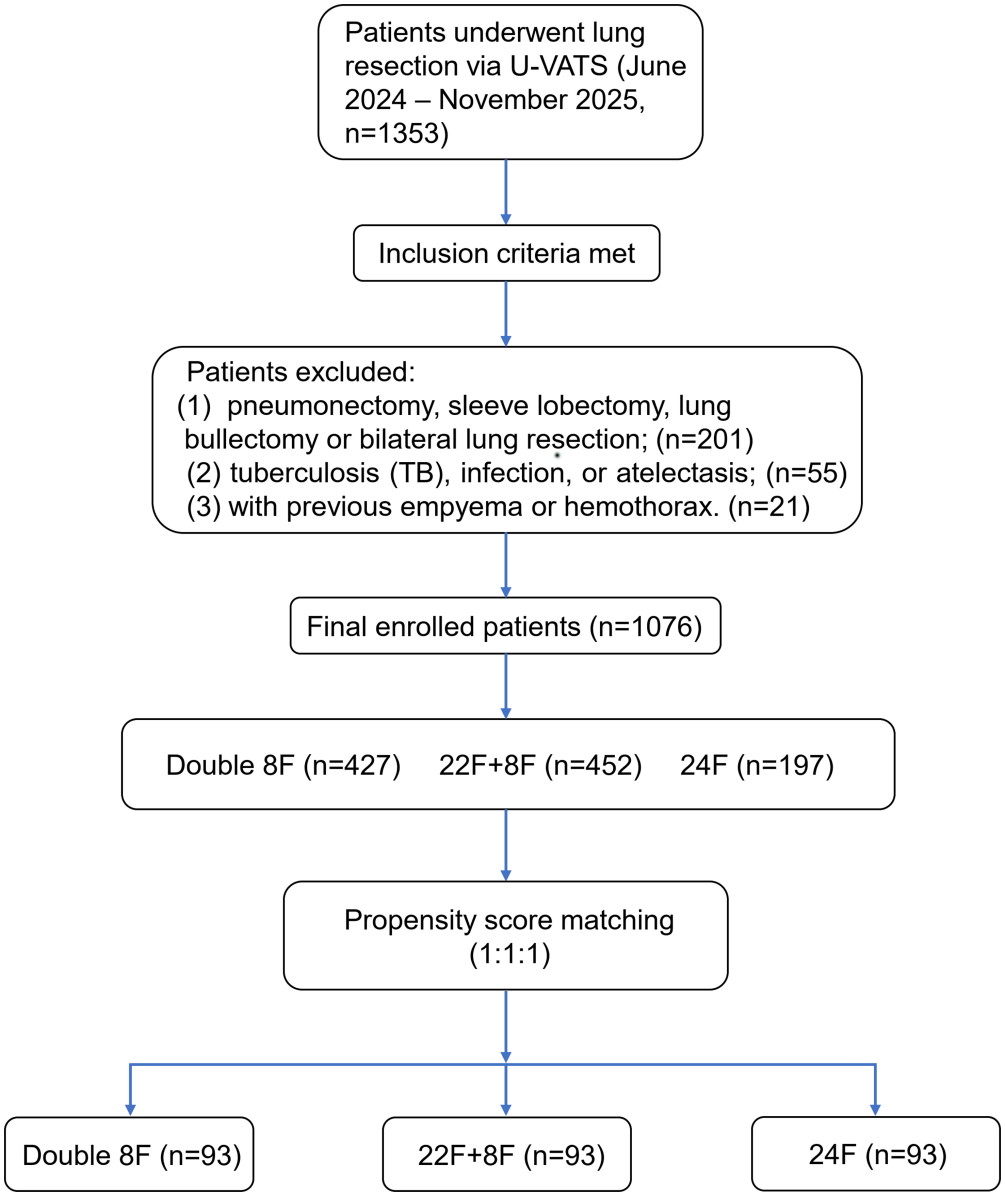
Patient flowchart of enrollment, inclusion, exclusion, and group allocation. U-VATS, Uniportal video-assisted thoracoscopic surgery.

The management of chest drainage following lung resection was the focus of this retrospective multicenter cohort study, which involved three Chinese participating institutions (the First Affiliated Hospital of Ningbo University, Sir Run Run Shaw Hospital of Zhejiang University and Ningbo Second Hospital). The study was approved by the Medical Ethics Committee of the First Affiliated Hospital of Ningbo University (2025-169A) and it was subsequently approved by the Institutional Review Boards (IRBs) of Sir Run Run Shaw Hospital (2025–1,176) and Ningbo Second Hospital (PJ-NBEY-KY-2025-160-01). All consecutive patients who underwent U-VATS for lung tumor at the three centers were retrospectively enrolled, with inclusion criteria and exclusion criteria strictly applied to ensure study homogeneity. Inclusion criteria were strictly defined as follows: (1) Patients diagnosed with lung tumor and scheduled for U-VATS, including lobectomy, segmentectomy, or wedge resection, between June 2024 and November 2025. (2) Complete perioperative medical records, demographic data, and follow-up data available for analysis. (3) Age ≥ 18 years. Patients with the following conditions were not included in the present study: (1) who underwent pneumonectomy, sleeve lobectomy, lung bullectomy or bilateral lung resection; (2) tuberculosis (TB), pulmonary infection, or atelectasis preoperative complications; (3) with previous empyema or hemothorax (Figure 1). First, all three Chinese hospitals performed comprehensive examinations at patient admission, including but not limited to imaging examinations (chest X-ray, chest CT), tuberculin skin test, and sputum culture. Final diagnosis was made based on a combination of these examinations and the patient’s clinical symptoms. Second, all patient data collected from the three centers were jointly reviewed and verified by reviewers Zhengfu He and Chenwei Li, both highly experienced thoracic surgeons.

**Figure 2:**
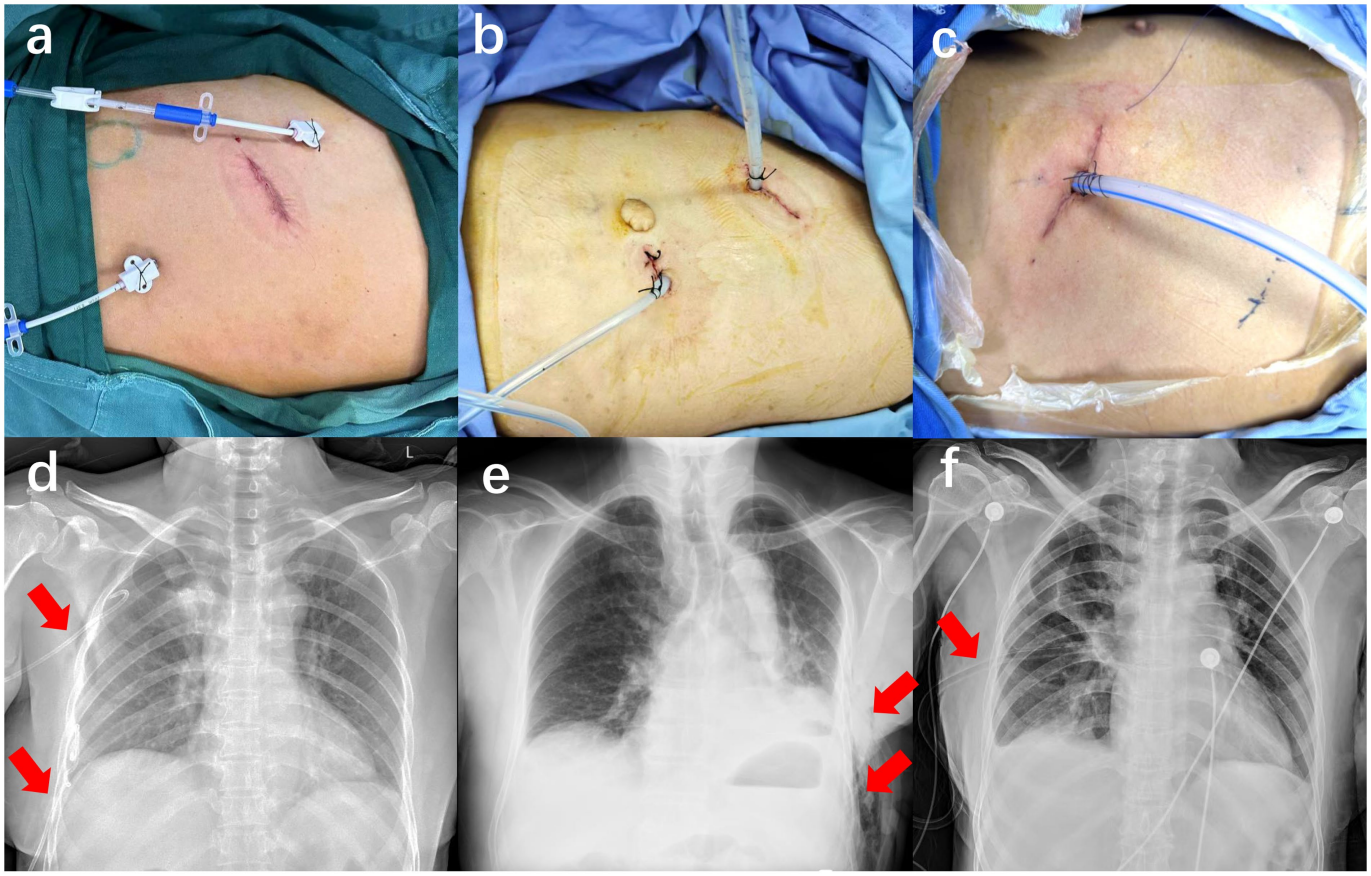
**(a)** Placement of two ultrafine 8F chest tubes, **(b)** Placement of one 22F chest tube and one 8F chest tube, and **(c)** Placement of one 24F chest tube after U-VATS for lung tumors. Postoperative thoracic cavity X-rays of patients treated with **(d)** two ultrafine 8F chest tubes, **(e)** one 22F chest tube plus one 8F chest tube, or **(f)** one 24F chest tube.

According to the above criteria, a total of 1,076 patients were enrolled. The patients had been classified for the type of drainage treatment and accordingly were allocated into three groups (Figure 2): 427 patients with double 8F ultrafine chest tube were allocated to the experimental group (double 8F group), whereas 452 patients with 22F chest tube and 8F chest tube and 197 patients with only 24F chest tube were allocated to the control group (22F + 8F group and 24F group). The study was approved by the ethics committees at each of the three hospitals. Given the retrospective nature of the study and the use of anonymized data, informed consent was waived.

### Perioperative management

The clinical variables evaluated in this study are summarized in Tables 1, 2. We classified patients into ever smokers and never-smokers based on their smoking history. Adhesion was defined as the intraoperative observation of fibrous bands between the chest wall and lung surface, involving one or more lobes. The choice and conversion of drainage methods were left to the preferences of surgeons, which varied across different hospitals. Notably, each participating center had no unified institutional clinical protocol for postoperative chest tube selection in U-VATS, and the drainage strategy was not pre-specified or associated with specific types of lung resection (including lobectomy, segmentectomy, and wedge resection); instead, the selection of double 8F ultrafine chest tubes, 22F + 8F combined chest tubes, or single 24F chest tubes was determined by the long-term clinical practice habits of individual attending thoracic surgeons at each center, with specific surgeons consistently using a single drainage strategy for all eligible U-VATS patients under their care.

**Table 1:** Baseline and intraoperative characteristics of patients enrolled in the study.

Variables	Total (*n* = 1,076)	Double 8F (*n* = 427)	22F + 8F (*n* = 452)	24F (*n* = 197)	Statistic	*p*
Age (years), M (Q₁, Q₃)	63.00 (53.00, 70.00)	60.00 (50.00,69.00)	65.00 (58.00,72.00)	63.00 (52.00,70.00)	*χ^2^* = 40.23#	**<0.001**
BMI (kg/m^2^), M (Q₁, Q₃)	22.84 (20.81,24.75)	22.88 (20.90,24.73)	22.80 (20.90,24.80)	22.68 (20.63,24.71)	*χ^2^* = 0.32#	0.852
Sex (male), *n* (%)	467 (43.40)	194 (45.43)	195 (43.14)	78 (39.59)	*χ^2^* = 1.89	0.388
Diabetes, *n* (%)	120 (11.15)	43 (10.07)	54 (11.95)	23 (11.68)	*χ^2^* = 0.85	0.655
Hypertension, *n* (%)	369 (34.29)	146 (34.19)	148 (32.74)	75 (38.07)	*χ^2^* = 1.73	0.421
Smoker status, *n* (%)					*χ^2^* = 12.40	0.002
Never-smoker	847 (78.72)	357 (83.61)	334 (73.89)	156 (79.19)		
Ever-smoker	229 (21.28)	70 (16.39)	118 (26.11)	41 (20.81)		
FEV₁% pred, *n* (%)					*χ^2^* = 19.60	0.003
FEV₁/FVC ≥ 70%	800 (74.35)	330 (77.28)	320 (70.80)	150 (76.14)		
FEV₁/FVC < 70%						
≥80%	157 (14.59)	43 (10.07)	89 (19.69)	25 (12.69)		
50% ~ 79%	103 (9.57)	49 (11.48)	35 (7.74)	19 (9.64)		
<50%	16 (1.49)	5 (1.17)	8 (1.77)	3 (1.52)		
TLC% pred, *n* (%)					*χ^2^* = 41.03	<0.001
≥80%	908 (84.39)	382 (89.46)	384 (84.96)	142 (72.08)		
60% ~ 79%	81 (7.53)	14 (3.28)	34 (7.52)	33 (16.75)		
50% ~ 59%	73 (6.78)	27 (6.32)	27 (5.97)	19 (9.64)		
<50%	14 (1.30)	4 (0.94)	7 (1.55)	3 (1.52)		
Pathology, *n* (%)					*χ^2^* = 13.01	0.001
Benign	132 (12.27)	70 (16.39)	38 (8.41)	24 (12.18)		
Malignant	944 (87.73)	357 (83.61)	414 (91.59)	173 (87.82)		
Metastatic cancer, n(%)	11 (1.02)	5 (1.17)	4 (0.88)	2 (1.02)	—	0.924
Type of resection, *n* (%)					*χ^2^* = 145.53	<0.001
Wedge resection	301 (27.97)	53 (12.41)	163 (36.06)	85 (43.15)		
Segmentectomy	386 (35.87)	235 (55.04)	98 (21.68)	53 (26.90)		
Lobectomy	389 (36.15)	139 (32.55)	191 (42.26)	59 (29.95)		
Location of resection, *n* (%)					*χ^2^* = 11.39	0.181
Left upper	262 (24.35)	100 (23.42)	106 (23.45)	56 (28.43)		
Left lower	177 (16.45)	67 (15.69)	71 (15.71)	39 (19.80)		
Right upper	315 (29.28)	142 (33.26)	127 (28.10)	46 (23.35)		
Right middle	97 (9.01)	39 (9.13)	44 (9.73)	14 (7.11)		
Right lower	225 (20.91)	79 (18.50)	104 (23.01)	42 (21.32)		
Adhesion, *n* (%)	368 (34.20)	137 (32.08)	176 (38.94)	55 (27.92)	*χ^2^* = 8.81	**0.012**

#, Kruskal-waills test; *χ^2^*, Chi-square test; −, Fisher exact; M, Median; Q₁, 1st Quartile; Q₃, 3st Quartile.

BMI, body mass index; FEV1, forced expiratory volume in the first second; FVC, forced vital capacity; FEV₁% pred, forced expiratory volume in 1 s percentage predicted; TLC% pred, total lung capacity percentage predicted; The bolded *p*-values in the table are all less than 0.05, indicating statistical significance.

**Table 2:** The outcomes between patients in different chest tube groups.

Variables	Total (*n* = 1,076)	Double 8F (*n* = 427)	22F + 8F (*n* = 452)	24F (*n* = 197)	Statistic	*p*
Hospital stay, M (Q₁, Q₃)	6.00 (5.00, 8.00)	7.00 (5.00,9.00)	6.00 (5.00,8.00)	6.00 (5.00,9.00)	*χ^2^* = 46.21#	**<0.001**
Drainage duration, M (Q₁, Q₃)	4.00 (3.00, 5.00)	4.00 (3.00,5.00)	4.00 (3.00,5.00)	3.00 (3.00,3.00)	*χ^2^* = 95.28#	**<0.001**
Drainage volume, M (Q₁, Q₃)						
POD1	110.00 (40.00, 200.00)	80.00 (20.00,140.00)	159.00 (99.50,246.25)	35.00 (0.00,120.00)	*χ^2^* = 196.93#	**<0.001**
POD2	110.00 (54.00, 195.00)	120.00 (70.00,200.00)	90.00 (40.75,175.00)	130.00 (60.00,230.00)	*χ^2^* = 21.87#	**<0.001**
POD3	60.00 (15.75, 115.00)	60.00 (0.00,117.50)	50.00 (13.00,90.00)	100.00 (50.00,155.00)	*χ^2^* = 39.26#	**<0.001**
Total	310.00 (189.50, 514.00)	320.00 (182.50,490.00)	315.50 (200.00,521.25)	270.00 (155.00,550.00)	*χ^2^* = 2.35#	0.309
NRS pain score, M (Q₁, Q₃)						
POD1	3.00 (3.00, 3.00)	3.00 (3.00,3.00)	3.00 (3.00,4.00)	3.00 (3.00,3.00)	*χ^2^* = 276.64#	**<0.001**
POD2	2.00 (2.00, 3.00)	2.00 (2.00,3.00)	3.00 (3.00,3.00)	2.00 (2.00,2.00)	*χ^2^* = 389.47#	**<0.001**
POD3	2.00 (1.00, 2.00)	2.00 (1.00,2.00)	2.00 (1.00,2.00)	1.00 (1.00,2.00)	*χ^2^* = 59.25#	**<0.001**
Complication, *n* (%)						
Pleural effusion	95 (8.83)	52 (12.18)	2 (0.44)	41 (20.81)	*χ^2^* = 80.59	**<0.001**
Infection	163 (15.15)	97 (22.72)	45 (9.96)	21 (10.66)	*χ^2^* = 31.60	**<0.001**
Air leakage	165 (15.33)	58 (13.58)	72 (15.93)	35 (17.77)	*χ^2^* = 2.03	0.363
Atelectasis	51 (4.74)	6 (1.41)	18 (3.98)	27 (13.71)	*χ^2^* = 46.16	**<0.001**
Post-extubation pneumothorax	30 (2.79)	5 (1.17)	5 (1.11)	20 (10.15)	*χ^2^* = 48.25	**<0.001**
Reintubation	4 (0.37)	3 (0.70)	0 (0.00)	1 (0.51)	—	0.189
Intrathoracic hemorrhage	27 (2.51)	4 (0.94)	4 (0.88)	19 (9.64)	*χ^2^* = 50.19	**<0.001**
Chylothorax	6 (0.56)	2 (0.47)	2 (0.44)	2 (1.02)	—	0.657

#, Kruskal-waills test; *χ^2^*, Chi-square test; −, Fisher exact; M, Median; Q₁, 1st Quartile; Q₃, 3st Quartile.

POD, postoperative day; NRS, numerical rating scale; The bolded *p*-values in the table are all less than 0.05, indicating statistical significance.

In the double 8F group, two 8F chest tubes (ABLE®; Baihe, Guangdong, China) were placed postoperatively. Both 8F ultrafine tubes were inserted through separate additional incisions. The inferior tube was positioned in the seventh to eighth intercostal space at the midaxillary line, while the superior tube was placed in the third intercostal space at the midaxillary line. In the 22F + 8F group, the 22F chest tube was inserted through the main surgical incision, whereas the 8F chest tube was placed via an additional incision. The 8F chest tube (ABLE®; Baihe, Guangdong, China) was positioned in the seventh to eighth intercostal space at the midaxillary line, and the 22F chest tube (MKL, Suzhou, China) was placed in the fourth to fifth intercostal space at the anterior axillary line. In the 24F group, a single 24F chest drainage tube (Pahsco, Taiwan, China) was inserted through the main surgical observation port at the seventh intercostal space following surgery. All chest tubes, regardless of being fine-bore or large-bore, were connected to a water-sealed drainage system (Figure 2 and Supplementary Figure S2).

Postoperatively, standard fluids were given, and electrocardiography (ECG) was carried out for ongoing vital sign monitoring. On the first postoperative day, the bedside chest X-ray, electrolyte levels, and blood gas analysis were evaluated. On the third day following surgery, computed tomography (CT) was done. This strategy enables early, sensitive, and objective detection of postoperative pneumothorax, pleural effusion, atelectasis, and other related complications. As a widely accepted routine in clinical practice across large medical institutions in China, it is crucial for reliably evaluating early postoperative recovery and guiding individualized chest tube management. Thoracic drainage nursing was used concurrently to make sure the drainage tube was clear.

A numeric rating scale (NRS) was used to measure postoperative pain, with each patient providing a subjective score ranging from 0 (no pain) to 10 (severe pain). On postoperative days (POD) 1, 2, and 3, pain scores were noted. Patients were instructed on how to cough properly before surgery, and they received additional training to guarantee successful postoperative implementation and accurate NRS scores during coughing. When the NRS score was higher than 3, further analgesia (50 mg intramuscular injection of Tramadol) was given, and the medication should be stopped as soon as the pain subsides. Total postoperative drainage volume, drainage duration, hospital stay, and postoperative complications (such as atelectasis, infection, air leakage, chest tube reinsertion, intrathoracic bleeding and chylothorax) were among the observation indicators. These indicators are observed and recorded jointly by doctors and nurses.

For patients with postoperative air leakage in all study groups, a standardized stepwise management protocol was implemented as follows: (1) Initial intervention: the chest tube was immediately connected to negative pressure suction to facilitate pleural re-expansion and air evacuation; (2) Subsequent management: in cases of persistent air leakage after initial intervention, high-concentration glucose was instilled into the thoracic cavity to induce pleural adhesion and seal the air leakage site. Additionally, intrathoracic drug administration was performed via the indwelling chest tube in accordance with sterile operative protocols, with tailored procedural steps for different chest tube calibers (ultrafine 8F tubes vs. large-bore tubes).

A postoperative infection is diagnosed if any of the following is met: (1) Follow-up chest CT/X-ray within a week post-op shows new pulmonary infiltrates. (2) Persistent or recurrent post-op temperature > 38.5 °C. (3) Post-op routine blood test: white blood cell (WBC) > 18 × 10^9^/L. (4) Post-op C-reactive protein (CRP) > 180 mg/L, with infection-related symptoms. A diagnosis of air leakage can be made if any of the following criteria is met: (1) Moderate or large pneumothorax (the lung tissue is compressed by more than 30%) was detected in the patient through imaging examinations (such as chest X-ray, CT). (2) Subcutaneous emphysema was documented, or visible bubbling in the water-seal chamber was noted at rest or with coughing in physicians’ or nurses’ progress notes and nursing records. Atelectasis was defined as involvement of at least one segment or lobe on chest X-ray or chest CT. Pleural effusion was defined as fluid accumulation reaching at least the upper margin of the 4th rib on chest radiograph or chest CT. Post-extubation pneumothorax was defined as pneumothorax occurring between chest tube removal and 30 days postoperatively (10). The diagnostic criteria for intrathoracic hemorrhage were defined as meeting at least one of the following: (1) Massive bloody drainage from the chest tube (> 1,000 mL within 24 h). (2) Receipt of red blood cell transfusion. (3) Re-thoracotomy or re-VATS for hemostasis due to bleeding. (4) Post-op hemoglobin < 90 g/L. Chylothorax was defined as a pleural triglyceride > 110 mg/dL or milky pleural drainage.

The following criteria were used to remove the chest tube: (1) a 24-h drainage volume of less than 100–200 mL; (2) no air leakage, chylothorax or intrathoracic bleeding; and (3) no pleural effusion or atelectasis symptoms. The following were the requirements for discharge: Inflammatory markers should be trending downward and approaching normal (WBC < 12 × 10^9^/L); fever should not be present (temperature ≤ 38 °C); and the chest drainage tube should be withdrawn.

### Statistical analysis

Descriptive statistical analyses were performed to summarize the demographic data of all enrolled subjects. For continuous variables (including age, body mass index, drainage duration, hospital stay, drainage volume/pain score on POD 1, 2, and 3, and total drainage volume), medians and interquartile ranges (IQR) were calculated. For categorical variables, frequencies and percentages were computed. To assess the significance of differences in continuous variables between the two cohorts, independent samples t-tests or Mann–Whitney U tests were applied, depending on data distribution. For categorical variables, chi-square (*χ^2^*) analysis or Fisher’s exact test was used, with the choice determined by sample size.

The multivariate linear regression model was used for multivariate analysis, incorporating variables with *p* < 0.05 from the univariate analysis. A forward-backward stepwise analysis method was adopted, with a *p*-value < 0.05 for variables used as both the inclusion and exclusion criteria, to identify the combination of factors that can most accurately predict the outcomes. Beta coefficients (*β*), standard errors (SE), and 95% confidence intervals (CI) were calculated to identify factors associated with drainage duration.

### Propensity score matching (PSM)

To minimize the potential confounding effects of baseline and intraoperative clinical heterogeneity (e.g., surgeon-specific drainage preferences, inter-center practice variations, differences in lung resection type and pleural adhesion status) and ensure valid and comparable perioperative outcome analyses among the double 8F, 22F + 8F, and single 24F groups, a 1:1:1 nearest-neighbor PSM was performed using the R package T-match (version 0.1.0).

1. *PSM model construction*: All baseline and intraoperative characteristics listed in Table 1 were included as covariates in the propensity score model, including age, BMI, sex, diabetes, hypertension, smoking status, forced expiratory volume in 1 s/forced vital capacity (FEV₁/FVC), total lung capacity percentage predicted (TLC% pred), pathological type (benign/malignant), metastatic status, type of lung resection (wedge/segmentectomy/lobectomy), resection location, and pleural adhesion.
2. *Matching criteria*: A strict caliper width of 0.2 (standard deviation of the logit of the propensity score) was set to reduce matching bias, which is a widely accepted threshold for PSM in surgical cohort studies.
3. *Balance assessment*: After matching, standardized mean differences (SMDs) were calculated to quantitatively evaluate covariate balance among the three groups; a threshold of SMD < 0.1 was considered to indicate adequate balance of all covariates. Love plots were used for visual balance diagnostics, which summarize the SMDs of all covariates before and after matching to intuitively demonstrate the reduction in intergroup heterogeneity.
4. *Matched cohort derivation*: The single 24F group had the smallest sample size (*n* = 197) among the three original cohorts; thus, the final matched cohort was determined by the size of the smallest group to maintain the 1:1:1 matching ratio. Unmatched patients with extreme propensity scores were excluded to ensure the validity and balance of the matched sample, resulting in a final matched cohort of 93 patients per group (279 total patients).

The length of hospital stay curve and chest tube removal curve were estimated using the Kaplan–Meier method. Subsequently, to further analyze pairwise differences between groups, multiple comparison correction (such as the Bonferroni method) was applied (Supplementary Tables S1, S2). All analyses were conducted using SPSS Statistics 26 (IBM Corporation, Armonk, NY, United States), with a two-tailed *p*-value < 0.05 deemed statistically significant.

## Result

The baseline of patients with different types of chest tubes is presented in Table 1 and the comparison of postoperative condition is displayed in Table 2.

In search of independent risk factors for drainage duration, univariate and multivariate regression analyses were conducted (Table 3 and Table 4). Many independent risk variables, including pleural adhesion (*β* = 0.31, 95% CI: 0.13 ~ 0.48, *p* < 0.01), infection (*β* = 0.44, 95% CI: 0.20 ~ 0.68, *p* < 0.01), air leakage (*β* = 0.83, 95% CI: 0.59 ~ 1.07, *p* < 0.01), intrathoracic hemorrhage (*β* = −1.32, 95% CI: −1.94 ~ −0.71, *p* < 0.01), drainage volume on POD1 (*β* = −0.01, 95% CI: −0.99 ~ −0.01, *p* < 0.01), POD2 (*β* = −0.01, 95% CI: −0.99 ~ −0.01, *p* < 0.01), and POD3 (*β* = −0.01, 95% CI: −0.99 ~ −0.01, *p* < 0.01), and total drainage volume (*β* = 0.01, 95% CI: 0.01 ~ 0.01, *p* < 0.01), are linked to drainage duration. Additionally, both the type of chest tube group and the type of lung resection exert an influence on drainage duration.

**Table 3:** Results of univariate linear regression with drainage duration as the outcome variable.

Variables	*β*	S. E	*t*	*p*	*β* (95% CI)
Chest tube group					
Double 8F					0.00 (Reference)
22F + 8F	−0.43	0.15	−2.80	**<0.01**	−0.43 (−0.73 ~ −0.13)
24F	−1.15	0.20	−5.87	**<0.01**	−1.15 (−1.53 ~ −0.77)
Age	0.03	0.01	5.35	**<0.01**	0.03 (0.02 ~ 0.04)
BMI	−0.06	0.02	−2.43	**0.02**	−0.06 (−0.10 ~ −0.01)
Sex (male)	0.71	0.14	5.03	**<0.01**	0.71 (0.43 ~ 0.98)
Diabetes	−0.10	0.22	−0.46	0.65	−0.10 (−0.54 ~ 0.34)
Hypertension	0.22	0.15	1.46	0.14	0.22 (−0.07 ~ 0.51)
Ever-smoker	0.91	0.17	5.38	**<0.01**	0.91 (0.58 ~ 1.25)
FEV₁% pred					
FEV₁/FVC ≥ 70%					0.00 (Reference)
FEV₁/FVC < 70%					
≥80%	0.56	0.20	2.83	**<0.01**	0.56 (0.17 ~ 0.95)
50% ~ 79%	1.06	0.24	4.42	**<0.01**	1.06 (0.59 ~ 1.52)
<50%	1.23	0.58	2.14	**0.03**	1.23 (0.10 ~ 2.36)
TLC% pred					
≥80%					0.00 (Reference)
60% ~ 79%	−0.17	0.27	−0.64	0.52	−0.17 (−0.69 ~ 0.35)
50% ~ 59%	1.15	0.28	4.12	**<0.01**	1.15 (0.60 ~ 1.69)
<50%	0.88	0.62	1.43	0.15	0.88 (−0.33 ~ 2.09)
Pathology (malignant)	0.32	0.21	1.48	0.14	0.32 (−0.10 ~ 0.74)
Metastatic cancer	−0.60	0.70	−0.85	0.39	−0.60 (−1.97 ~ 0.78)
Type of resection					
Wedge resection					0.00 (Reference)
Segmentectomy	0.63	0.17	3.71	**<0.01**	0.63 (0.30 ~ 0.96)
Lobectomy	1.63	0.17	9.61	**<0.01**	1.63 (1.30 ~ 1.97)
Location of resection					
Left upper					0.00 (Reference)
Left lower	−0.23	0.22	−1.03	0.30	−0.23 (−0.67 ~ 0.21)
Right upper	−0.18	0.19	−0.91	0.36	−0.18 (−0.55 ~ 0.20)
Right middle	−0.12	0.27	−0.43	0.67	−0.12 (−0.66 ~ 0.42)
Right lower	−0.05	0.21	−0.25	0.80	−0.05 (−0.46 ~ 0.36)
Adhesion	0.67	0.15	4.55	**<0.01**	0.67 (0.38 ~ 0.96)
Drainage volume					
POD1	0.01	0.00	8.84	**<0.01**	0.01 (0.01 ~ 0.01)
POD2	0.01	0.00	9.73	**<0.01**	0.01 (0.01 ~ 0.01)
POD3	0.01	0.00	14.47	**<0.01**	0.01 (0.01 ~ 0.01)
Total	0.01	0.00	31.37	**<0.01**	0.01 (0.01 ~ 0.01)
NRS pain score					
POD1	−0.08	0.13	−0.65	0.51	−0.08 (−0.33 ~ 0.16)
POD2	0.16	0.10	1.69	0.09	0.16 (−0.03 ~ 0.36)
POD3	0.29	0.09	3.04	**<0.01**	0.29 (0.10 ~ 0.47)
Complication					
Pleural effusion	1.67	0.24	6.86	**<0.01**	1.67 (1.19 ~ 2.14)
Infection	1.71	0.19	9.03	**<0.01**	1.71 (1.34 ~ 2.08)
Air leakage	2.30	0.18	12.63	**<0.01**	2.30 (1.95 ~ 2.66)
Atelectasis	1.63	0.33	4.98	**<0.01**	1.63 (0.99 ~ 2.27)
Post-extubation pneumothorax	0.88	0.43	2.07	**0.04**	0.88 (0.05 ~ 1.72)
Reintubation	3.10	1.15	2.69	**<0.01**	3.10 (0.84 ~ 5.36)
Intrathoracic hemorrhage	2.62	0.44	5.92	**<0.01**	2.62 (1.75 ~ 3.49)
Chylothorax	3.95	0.94	4.21	**<0.01**	3.95 (2.11 ~ 5.78)

CI, Confidence interval.

BMI, body mass index; FEV1, forced expiratory volume in the first second; FVC, forced vital capacity; FEV₁% pred, forced expiratory volume in 1 s percentage predicted; TLC% pred, total lung capacity percentage predicted; POD, postoperative day; NRS, numerical rating scale; The bolded *p*-values in the table are all less than 0.05, indicating statistical significance.

**Table 4:** Results of multivariate linear regression with drainage duration as the outcome variable.

Variables	*β*	S. E	*t*	*p*	*β* (95% CI)
Intercept	3.03	0.24	12.50	**<0.01**	3.03 (2.56 ~ 3.51)
Chest tube group					
Double 8F					0.00 (Reference)
22F + 8F	−0.39	0.11	−3.48	**<0.01**	−0.39 (−0.60 ~ −0.17)
24F	−0.93	0.13	−7.05	**<0.01**	−0.93 (−1.18 ~ −0.67)
Age	0.01	0.00	1.91	0.06	0.01 (−0.00 ~ 0.01)
Ever-smoker	0.20	0.11	1.82	0.07	0.20 (−0.01 ~ 0.41)
Type of resection					
Wedge resection					0.00 (Reference)
Segmentectomy	0.14	0.11	1.22	0.22	0.14 (−0.09 ~ 0.37)
Lobectomy	0.51	0.12	4.37	**<0.01**	0.51 (0.28 ~ 0.74)
Adhesion	0.31	0.09	3.42	**<0.01**	0.31 (0.13 ~ 0.48)
Drainage volume					
POD1	−0.01	0.00	−6.65	**<0.01**	−0.01 (−0.99 ~ −0.01)
POD2	−0.01	0.00	−11.03	**<0.01**	−0.01 (−0.99 ~ −0.01)
POD3	−0.01	0.00	−5.44	**<0.01**	−0.01 (−0.99 ~ −0.01)
Total	0.01	0.00	29.09	**<0.01**	0.01 (0.01 ~ 0.01)
NRS pain score					
POD3	0.09	0.06	1.48	0.14	0.09 (−0.03 ~ 0.20)
Complication, *n* (%)					
Infection	0.44	0.12	3.60	**<0.01**	0.44 (0.20 ~ 0.68)
Air leakage	0.83	0.12	6.68	**<0.01**	0.83 (0.59 ~ 1.07)
Reintubation	1.61	0.69	2.32	**0.02**	1.61 (0.25 ~ 2.97)
Intrathoracic hemorrhage	−1.32	0.31	−4.23	**<0.01**	−1.32 (−1.94 ~ −0.71)

CI, Confidence interval; POD, postoperative day; NRS, numerical rating scale; The bolded *p*-values in the table are all less than 0.05, indicating statistical significance.

**Figure 3:**
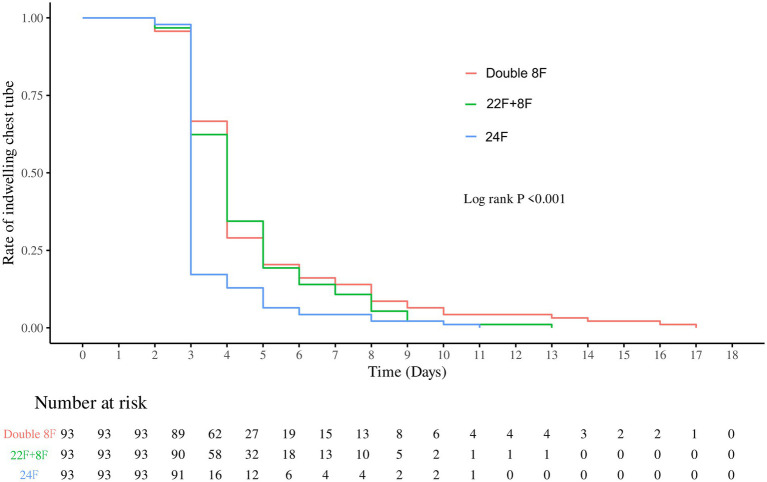
Chest tube indwelling rate stratified by chest tube group after propensity score matching.

**Figure 4:**
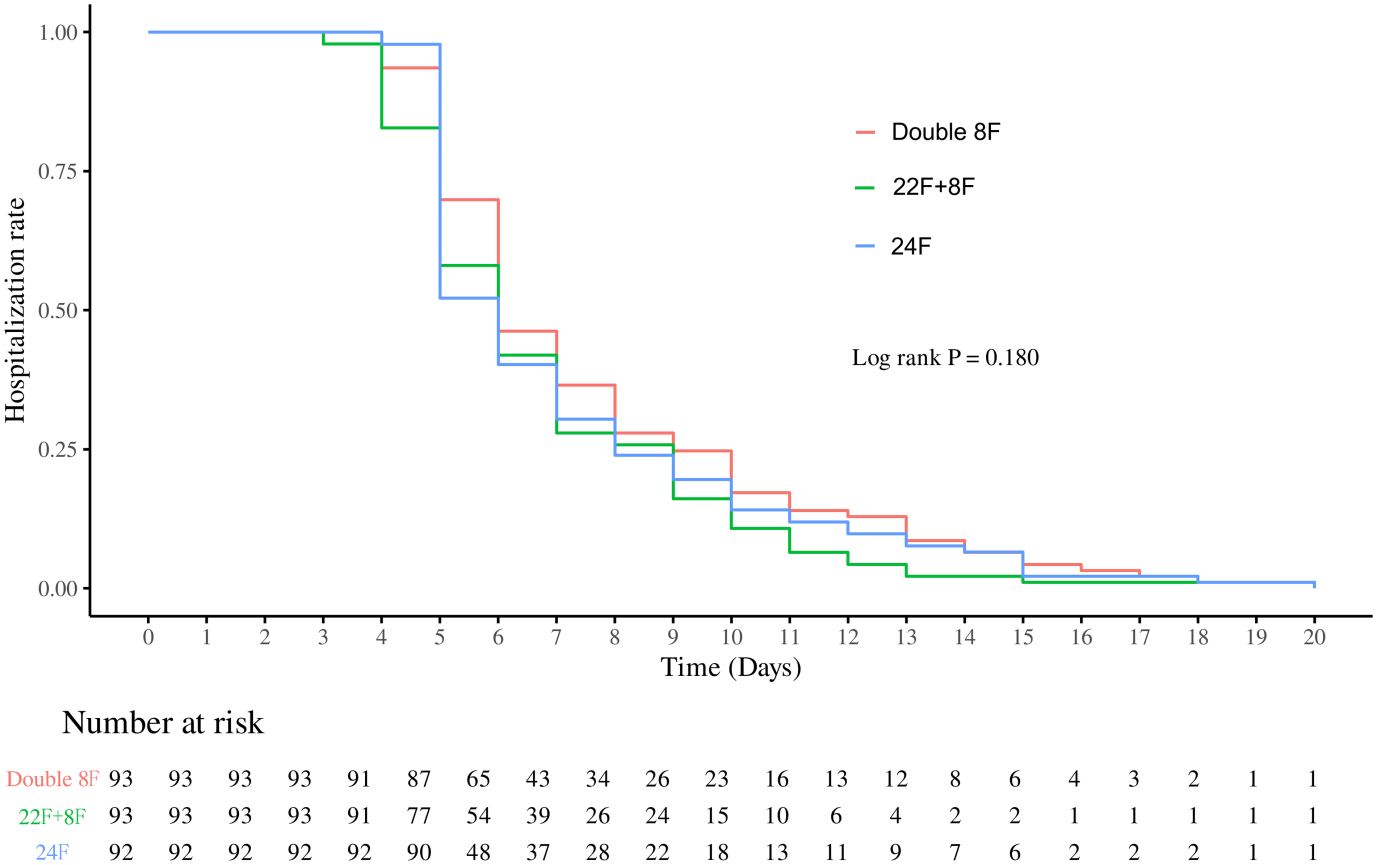
Hospitalization rate stratified by chest tube group after propensity score matching.

**Figure 5:**
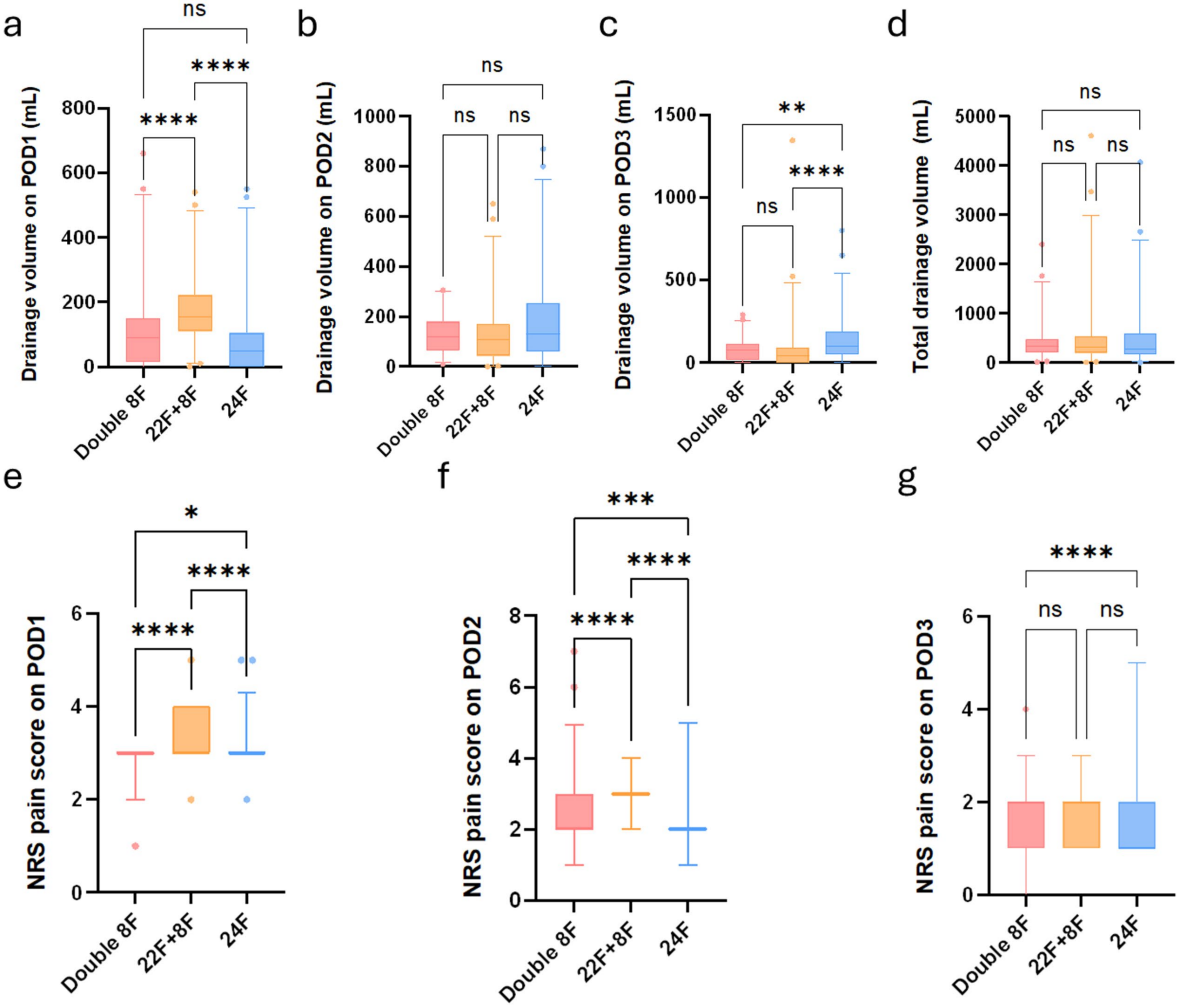
Boxplots show comparison of drainage volume among three chest tube groups after propensity score matching. **(a)** Drainage volume on POD1. **(b)** Drainage volume on POD2. **(c)** Drainage volume on POD3. **(d)** Total drainage volume. **(e)** NRS pain score on POD1. **(f)** NRS pain score on POD2. **(g)** NRS pain score on POD3. NRS, numerical rating scale. POD, postoperative day. (NS, not significant; *p < 0.05; **p < 0.01; ***p < 0.001; ****p < 0.0001; NRS, numerical rating scale).

**Figure 6:**
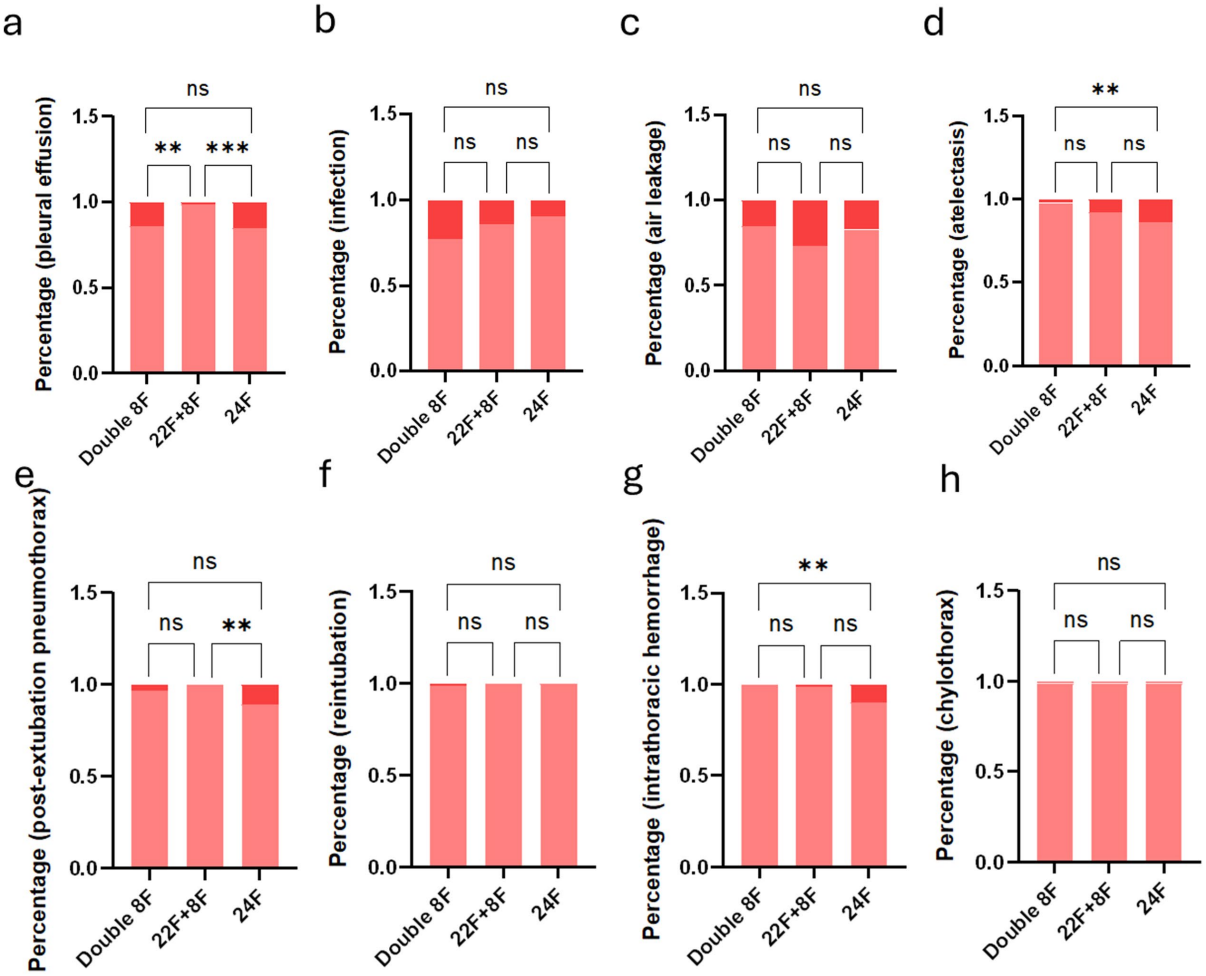
Comparison of the incidence of complications among three chest tube groups after propensity score matching. **(a)** Proportion of pleural effusion cases by group (three groups). **(b)** Proportion of infection cases by group (three groups). **(c)** Proportion of air leakage cases by group (three groups). **(d)** Proportion of atelectasis cases by group (three groups). **(e)** Proportion post-extubation pneumothorax of cases by group (three groups). **(f)** Proportion of reintubation cases by group (three groups). **(g)** Proportion of intrathoracic hemorrhage cases by group (three groups). **(h)** Proportion of chylothorax cases by group (three groups). Red: Presence of this complication; Pink: Absence of this complication. (NS, not significant; *p < 0.05; **p < 0.01; ***p < 0.001; ****p < 0.0001).

After 1:1:1 PSM, we gathered the results of 279 patients (93 pairs) with double 8F group, 22F + 8F group and 24F group. No statistically significant differences were observed in any covariate between the three matched groups (all *p* > 0.05), indicating that the PSM effectively eliminated intergroup clinical heterogeneity and minimized potential confounding biases. Differences between each pair of groups are shown in Figure 3–6, Supplementary Tables S1, S2.

### Perioperative indicators among the three matched groups

After PSM, significant intergroup differences were observed in multiple key perioperative outcomes (Table 5), and the detailed pairwise intergroup comparisons (with Bonferroni correction for multiple comparisons) were further validated in Supplementary Table S1, with the results as follows:

**Table 5:** Postoperative outcomes in 1:1:1 PSM.

Variables	Double 8F (*n* = 93)	22F + 8F (*n* = 93)	24F (*n* = 93)	Statistic	*p*
Hospital stay, M (Q₁, Q₃)	6.00 (5.00,9.00)	6.00 (5.00,9.00)	6.00 (5.00,8.00)	*χ^2^* = 3.65#	0.161
Chest tube duration, M (Q₁, Q₃)	4.00 (3.00,5.00)	4.00 (3.00,5.00)	3.00 (3.00,3.00)	*χ^2^* = 38.52#	**<0.001**
Drainage volume, M (Q₁, Q₃)					
POD1	90.00 (15.00,150.00)	155.00 (110.00,219.00)	50.00 (0.00,100.00)	*χ^2^* = 48.00#	**<0.001**
POD2	120.00 (65.00,180.00)	107.00 (45.00,170.00)	130.00 (60.00,250.00)	*χ^2^* = 4.54#	0.103
POD3	75.00 (20.00,110.00)	44.00 (2.00,92.00)	100.00 (50.00,180.00)	*χ^2^* = 23.12#	**<0.001**
Total	325.00 (210.00,470.00)	319.00 (197.00,522.00)	270.00 (170.00,590.00)	*χ^2^* = 0.28#	0.869
NRS pain score, M (Q₁, Q₃)					
POD1	3.00 (3.00,3.00)	3.00 (3.00,4.00)	3.00 (3.00,3.00)	*χ^2^* = 71.90#	**<0.001**
POD2	2.00 (2.00,3.00)	3.00 (3.00,3.00)	2.00 (2.00,2.00)	*χ^2^* = 111.93#	**<0.001**
POD3	2.00 (1.00,2.00)	2.00 (1.00,2.00)	1.00 (1.00,2.00)	*χ^2^* = 20.37#	**<0.001**
Complication					
Pleural effusion	13 (13.98)	1 (1.08)	14 (15.05)	—	**<0.001**
Infection	21 (22.58)	13 (13.98)	9 (9.68)	—	0.052
Air leakage	14 (15.05)	25 (26.88)	16 (17.20)	—	0.113
Atelectasis	2 (2.15)	7 (7.53)	13 (13.98)	—	**0.011**
Post-extubation pneumothorax	3 (3.23)	0 (0.00)	10 (10.75)	—	**0.001**
Reintubation	1 (1.08)	0 (0.00)	0 (0.00)	—	1.000
Intrathoracic hemorrhage	0 (0.00)	1 (1.08)	9 (9.68)	—	**<0.001**
Chylothorax	1 (1.08)	1 (1.08)	1 (1.08)	—	1.000

#, Kruskal-waills test; *χ^2^*, Chi-square test; −, Fisher exact; M, Median; Q₁, 1st Quartile; Q₃, 3st Quartile.

PSM, propensity score matching; POD, postoperative day; NRS, numerical rating scale.

The bolded *p*-values in the table are all less than 0.05, indicating statistical significance.

Drainage duration and hospital stay: The overall difference in drainage duration among the three groups was statistically significant (*p* < 0.0001). Pairwise comparison showed the single 24F group had the shortest drainage duration, with significant differences compared with both the double 8F group and the 22F + 8F group (both *p* < 0.0001), while there was no significant difference in drainage duration between the double 8F group and the 22F + 8F group (*p* > 0.9999) (Figure 3 and Supplementary Table S1). No statistically significant overall difference in hospital stay was found among the three groups (*p* = 0.1800), and thus no pairwise *post hoc* comparisons were conducted (Figure 4 and Supplementary Table S1).

Drainage volume: Significant overall differences were observed in drainage volume on POD1 and POD3 (both *p* < 0.0001), while no significant overall differences were found in POD2 drainage volume and total drainage volume (*p* = 0.1033 and *p* = 0.8690, respectively). For POD1 drainage volume, the double 8F group had a significantly lower volume than the 22F + 8F group (*p* < 0.0001), and the 22F + 8F group had a significantly higher volume than the 24F group (*p* < 0.0001), with no significant difference between the double 8F group and the 24F group (*p* = 0.1317). For POD3 drainage volume, no significant difference was found between the double 8F group and the 22F + 8F group (*p* = 0.4739), while both double 8F and 22F + 8F groups had significantly lower drainage volume than the 24F group (double 8F vs. 24F, *p* = 0.0032; 22F + 8F vs. 24F, *p* < 0.0001) (Figure 5 and Supplementary Table S1).

Postoperative pain (NRS score): Significant overall differences in NRS pain scores were observed on POD1, POD2 and POD3 (all p < 0.0001). Pairwise comparisons showed the double 8F group had significantly lower NRS pain scores than the 22F + 8F group on all 3 days (all *p* < 0.0001). The single 24F group had significantly lower pain scores than both the double 8F group and the 22F + 8F group on POD1 (double 8F vs. 24F, *p* = 0.0169), POD2 (double 8F vs. 24F, *p* = 0.0008) and POD3 (double 8F vs. 24F, *p* < 0.0001). For POD3 pain score, no significant differences were found between the double 8F group and the 22F + 8F group (*p* = 0.0694), nor between the 22F + 8F group and the 24F group (*p* = 0.0748) after correction (Figure 5 and Supplementary Table S1).

### Postoperative complications among the three matched groups

After PSM, significant overall differences were observed in the incidence of pleural effusion, atelectasis, post-extubation pneumothorax and intrathoracic hemorrhage among the three groups (all *p* < 0.05), while no significant overall differences were found in infection, air leakage, reintubation and chylothorax (all *p* > 0.05). Detailed pairwise intergroup comparisons (Bonferroni-corrected) for complications are shown in Supplementary Table S2:

Pleural effusion: The overall difference was significant (*p* = 0.0005). Pairwise comparison showed the 22F + 8F group had the lowest incidence, with significant differences compared with both the double 8F group (*p* = 0.0036) and the 24F group (*p* = 0.0018), while no significant difference was found between the double 8F group and the 24F group (*p* > 0.9999) (Figure 6 and Supplementary Table S2).

Atelectasis: The overall difference was significant (*p* = 0.0111). Pairwise comparison showed the double 8F group had a significantly lower incidence than the 24F group (*p* = 0.0159), while no significant differences were found between the double 8F group and the 22F + 8F group (*p* = 0.5070), nor between the 22F + 8F group and the 24F group (*p* = 0.7083) (Figure 6 and Supplementary Table S2).

Post-extubation pneumothorax: The overall difference was significant (*p* = 0.0013). The 22F + 8F group had the lowest incidence (0%), with a significant difference compared with the 24F group (*p* = 0.0045), while no significant difference was found between the double 8F group and the 22F + 8F group (*p* = 0.7377), nor between the double 8F group and the 24F group (*p* = 0.2430) (Figure 6 and Supplementary Table S2).

Intrathoracic hemorrhage: The overall difference was significant (*p* = 0.0008). The double 8F group had the lowest incidence (0%), with a significant difference compared with the 24F group (*p* = 0.0096), while no significant differences were found between the double 8F group and the 22F + 8F group (*p* > 0.9999), and the difference between the 22F + 8F group and the 24F group was not statistically significant after correction (*p* = 0.0549) (Figure 6 and Supplementary Table S2).

No significant intergroup differences: For infection (*p* = 0.0524), air leakage (*p* = 0.1130), reintubation (*p* > 0.9999) and chylothorax (*p* > 0.9999) (Figure 6 and Supplementary Table S2), no significant overall differences were observed among the three groups, and thus no pairwise *post hoc* comparisons were conducted.

## Discussion

In recent years, minimally invasive surgery has gained widespread acceptance due to the promotion and use of the notion of ERAS (11). Therefore, clinicians are increasingly concerned about how the use of thoracic drainage tubes affects patients’ postoperative recovery.

According to the latest expert consensus of the Society of Thoracic Surgeons, the quantity, size, and material of drainage tubes all affect the postoperative drainage effect (12). After univariate and multivariate analyses, this study also found that the use of chest tubes of different sizes and quantities postoperatively affects drainage duration. Additionally, it identified that factors such as surgical approach, pleural adhesions, postoperative drainage volume, postoperative infection, postoperative air leakage, and intrathoracic hemorrhage are also associated with drainage duration.

Following 1:1:1 propensity score matching, the association between chest tube size/quantity and drainage duration remained significant. The study observed that the drainage duration in the single chest tube group (24F group) was shorter than that in the two double chest tube groups (double 8F group and 22F + 8F group). A study by You J et al., which evaluated 11 randomized trials, found that the drainage duration in the single chest tube group was significantly shorter than that in the double chest tube group (13). This finding was further confirmed by studies conducted by Zhang X et al. and Zhou D et al. (7, 14)—likely due to more aggressive chest tube removal thresholds routinely adopted by clinical surgeons for patients managed with a single large-bore tube. The study also found that the double 8F chest tube was comparable to the other two drainage methods in terms of hospital stay.

Routine indwelling of thoracic drainage tubes is required after U-VATS. However, these tubes can compress intercostal nerves and stimulate the diaphragm, leading to postoperative pain that hinders patient recovery. Song Y et al. compared the drainage performance of 8F ultrafine pigtail tubes and conventional 24F thoracic drainage tubes in patients who underwent U-VATS. Their findings demonstrated that pigtail tubes were more effective in mitigating postoperative pain (15). Similarly, a study by Xu Y et al. indicated that the 12F pigtail tube reduced the severity of cough-related pain after surgery compared with the conventional 20F thoracic tube (9). In our study, 8F group exhibited significantly lower pain scores than 22F + 8F group on the first and second postoperative days, with no statistically significant difference observed on the third day. This suggests that when the same number of thoracic tubes are used for postoperative drainage, the smaller caliber 8F tube offers greater advantages in reducing postoperative pain. We propose that ultrafine chest tubes do not compress the intercostal nerves, and their soft texture produces minimal stimulation to the diaphragm—two key mechanistic factors that align with the findings of Xu et al. (9) and Song et al. (15) and collectively contribute to a clinically meaningful reduction in postoperative pain for patients. Notably, unlike these single-center RCTs that evaluated single 8F/12F tubes, our multicenter study validates that this pain relief advantage is retained even with double 8F tube placement, eliminating clinical concerns that dual-tube placement may increase thoracic irritation and pain. Compared with a single 24F thoracic tube, the double 8F thoracic tubes were associated with lower pain scores on the first postoperative day but higher pain scores on the second and third postoperative days.

On the first postoperative day, the drainage volumes of both the double 8F chest tube group and the single 24F chest tube group were significantly lower than that of the 22F group. On the third postoperative day, the drainage volumes of the double-tube groups (8F and 22F) were significantly less than that of the single 24F chest tube group. These results indicate that the double 8F drainage tubes have an advantage in reducing drainage volume in the early postoperative period. Two potential factors may account for this finding. First, the placement of fine drainage tubes does not require additional manipulation or cause further trauma to the surgical incision, thereby reducing intraoperative bleeding and postoperative exudation. Second, the fine tubes exhibit good flexibility, and their pigtail design prevents kinking. Postoperatively, they can be coiled at the costophrenic angle—the lowest point of the pleural cavity—ensuring adequate drainage. Additionally, the ultrafine chest tubes have a smooth inner wall and strong anticoagulation capacity, which enables unobstructed drainage. Studies by Xu Y et al. and Song Y et al. both noted that the total drainage volume of a single pigtail tube was lower than that of a single large-bore tube (9, 15). However, in our study, no significant difference in total drainage volume was observed among the three groups, including the double 8F group. This discrepancy is attributable to two key study design differences: (1) tube number: previous studies compared single ultrafine tubes with single large-bore tubes, while our study evaluated double 8F ultrafine tubes—the dual-tube placement ensures more comprehensive thoracic drainage, offsetting the potential reduction in single-tube drainage volume and resulting in a total drainage volume comparable to conventional methods; (2) patient population: our multicenter cohort included a higher proportion of segmentectomy patients (55.04% in the double 8F group) compared with the single-center RCTs (9, 15), which mainly enrolled lobectomy and wedge resection patients. Segmentectomy involves a larger operative field and more pleural dissection, leading to slightly increased pleural exudation that contributes to similar total drainage volume.

There were also significant differences in the incidence of postoperative complications across the three drainage methods, with the double 8F chest tubes demonstrating notable advantages in preventing postoperative atelectasis and intrathoracic hemorrhage (both *p* < 0.05), and no significant increase in infection risk compared with the other two drainage strategies (*p* = 0.052), further supporting the safety of the double 8F ultrafine chest tube strategy. The potential mechanisms underlying this observation are as follows: (1) Ultrafine chest tubes exert minimal compression on lung lobes and the diaphragm, which in turn reduces the risk of intrathoracic hemorrhage. (2) Patients in the ultrafine chest tube group experience less prominent pain, enabling earlier initiation of pulmonary function exercises—this facilitates more effective lung recruitment. Furthermore, the double 8F chest tubes were comparable to the other two groups in preventing primary postoperative pneumothorax, reintubation, post-extubation pneumothorax and chylothorax. This aligns with findings from Xu Y et al. and Song Y et al. (9, 15), who reported no significant difference in the incidence of infectious and air leakage complications between fine-bore and large-bore tubes in single-center RCTs. Our multicenter study further validates this safety profile in a real-world heterogeneous cohort, and additionally demonstrates a novel advantage of double 8F tubes in reducing atelectasis and intrathoracic hemorrhage—an outcome not reported in previous single-center studies, likely due to their smaller sample sizes and limited ability to detect differences in rare complications. In addition, this study found that 22F + 8F group outperformed the single 24F chest tube in preventing specific postoperative complications—a result also noted in research by Yang Z et al. They proposed that, compared with a single 26F chest tube, the combined use of a 26F and an 8F chest tube after single-port thoracoscopic surgery could lower the incidence of postoperative complications (16). However, their study did not investigate the double 8F chest tube configuration employed in the present research.

Although double 8F ultrafine chest drainage tubes achieved equivalent efficacy in air leakage drainage relative to 22F + 8F combined tubes and single 24F chest tubes, intrathoracic injection of high-concentration glucose via 8F ultrafine tubes offers a more facile and sterility-enhanced procedural approach for patients with persistent air leakage. This distinctive advantage may translate into additional clinical benefits for the management of persistent postoperative air leakage (15). Additionally, Xu Y et al. noted that fine-bore tubes exert a positive effect on wound healing, as indicated by a significantly lower incidence of wound complications and reduced severity of such complications in the fine-bore tube group compared with the large-bore tube group (9). Notably, this study did not incorporate an assessment of wound conditions following chest tube insertion.

In the original unmatched cohort, the double 8F group showed a numerically higher infection rate compared with the 22F + 8F and 24F groups (Table 2), but this finding was not statistically significant after 1:1:1 PSM (*p* = 0.052, Table 5), confirming it was driven by baseline clinical heterogeneity rather than the drainage strategy itself. A key potential explanation for the initial numerical difference is the baseline distribution of clinical characteristics in the unmatched cohort: the double 8F group had a higher proportion of patients with impaired lung function (lower TLC% pred and FEV1/FVC, Table 1) and a higher rate of segmentectomy (55.04%, Table 1)—a surgical procedure with a relatively larger intrapleural operative field compared with wedge resection. Impaired preoperative lung function is a well-recognized risk factor for postoperative pulmonary infection (17), and a larger operative field may slightly increase the risk of mild pleural inflammation, which was classified as infection per the study’s strict diagnostic criteria. Additionally, the study’s infection definition included mild inflammatory changes with elevated CRP (>180 mg/L) and white blood cell count (>18 × 10^9^/L) (see Methods), which may have captured more mild, self-limiting inflammatory responses in the double 8F group rather than severe clinical infections requiring targeted antimicrobial therapy. Importantly, in the PSM-matched cohort—where baseline lung function, surgical procedure, and other confounding factors were balanced—there was no significant difference in infection rate across the three groups, which confirms that the double 8F drainage strategy does not increase the risk of postoperative infection. This finding is consistent with previous studies that reported no difference in infectious complication rates between ultrafine pigtail catheters and large-bore chest tubes after U-VATS (9, 15).

The persistently higher incidence of pleural effusion in the double 8F group after PSM is attributable to the intrinsic physiological properties of ultrafine chest tubes combined with the surgical characteristics of the cohort, rather than insufficient drainage efficacy of the tubes. The double 8F group had a predominance of patients undergoing segmentectomy, a procedure involving more extensive pleural dissection that inherently induces greater physiological pleural exudation. Furthermore, the ultrafine 8F caliber, in contrast to the large-bore 24F/22F tubes, exerts minimal mechanical irritation on the pleural surface and lacks the “forced rapid drainage” effect of larger tubes. This results in a more gradual and physiological absorption of pleural effusion in the double 8F group, rather than an acute evacuation of exudate. Notably, all cases of pleural effusion in the double 8F group were mild and asymptomatic, after propensity score matching, only one patient with pleural effusion in the double 8F group required chest tube reinsertion for therapeutic drainage, and there was no statistically significant difference in the incidence of chest tube reinsertion among the three groups (Table 5, *p* = 1.000). This distinguishes the effusions in the double 8F group from clinically significant, symptomatic pleural effusions and confirms that the higher incidence represents a benign physiological finding rather than a clinically adverse outcome associated with the double 8F drainage strategy.

The single 24F group exhibited favorable performance in several perioperative indicators in the PSM-matched cohort, including lower drainage volume on POD1 and reduced NRS pain score on POD3 (Table S1). However, these advantages lack substantial clinical significance and did not translate into meaningful improvements in key clinical outcomes: the lower POD1 drainage volume was attributed to the more aggressive chest tube removal threshold routinely adopted for single large-bore tubes in clinical practice, with no corresponding difference in total drainage volume (*p* = 0.869) or hospital stay (*p* = 0.161) observed among the three groups. Additionally, the superior pain relief in the 24F group was only noted on POD3, while the double 8F group demonstrated significantly lower pain scores during POD1-POD2 (all *p* < 0.05)—the critical window for postoperative acute pain and ERAS implementation, where pain relief directly impacts patients’ tolerance for early functional exercises (e.g., deep breathing, effective coughing) and reduces the risk of exercise-related complications. The minor pain difference on POD3 did not reach the minimally clinically important difference (MCID) for postoperative pain in thoracic surgery, and thus had no meaningful impact on long-term postoperative recovery.

A modest 1-point reduction in the numerical rating scale (NRS) pain score (0–10) was observed in the double 8F group on postoperative days (POD) 1–3, which may appear trivial in statistical terms but carries substantial clinical meaning for patients undergoing uniportal video-assisted thoracoscopic surgery (U-VATS). First, the NRS is a validated subjective pain assessment tool in thoracic surgery, and a 1-point change in the moderate pain range (NRS 3–4, the baseline in this study) is recognized as a minimally clinically important difference (MCID) for postoperative acute pain (18). For U-VATS patients, even a small reduction in pain intensity directly improves their tolerance for early functional exercises (e.g., deep breathing, effective coughing, ambulation), which are the cornerstones of preventing postoperative complications such as atelectasis and pulmonary infection. Second, the pain reduction in the double 8F group was most pronounced on POD1, the period of peak postoperative pain and the critical window for ERAS implementation. Reduced pain on POD1 reduces the need for rescue analgesia (e.g., tramadol intramuscular injection in this study), minimizes the adverse effects of analgesics (e.g., nausea, vomiting, somnolence), and accelerates the recovery of gastrointestinal and respiratory function—key ERAS goals. Third, the sustained pain relief on POD2–3 further promotes continuous postoperative rehabilitation, reducing the duration of bed rest and the risk of immobilization-related complications (e.g., deep vein thrombosis), which indirectly contributes to shortened hospital stay and improved patient-reported outcomes (PROs).

The double 8F group exhibited significantly lower drainage volume on POD1 and POD3 compared with the 22F + 8F group, and while this difference did not translate into a reduction in total drainage volume, the reduction in early drainage volume has important clinical implications for postoperative thoracic management. First, the reduction in early postoperative drainage volume (particularly on POD1) in the double 8F group is closely associated with diminished intraoperative bleeding and reduced pleural exudation, a finding underpinned by both physiological characteristics of ultrafine chest tubes and clinical procedural considerations. Physiologically, the ultrafine 8F caliber (far smaller than conventional large-bore tubes) and its soft, flexible material minimize iatrogenic trauma to intercostal vessels, pleural parenchyma and surgical dissection sites during tube insertion—anatomical structures that are major sources of intraoperative bleeding and postoperative pleural exudation in thoracic surgery. Clinically, the placement of double 8F ultrafine tubes requires no additional incisions or aggressive tissue dissection beyond the U-VATS port, and the pigtail distal design avoids tube-induced laceration of the visceral or parietal pleura during positioning. Collectively, these minimally invasive properties of the double 8F ultrafine tube system reduce the degree of acute tissue injury and hemostatic disruption intraoperatively, which directly translates to less sanguineous drainage and reduced inflammatory pleural exudation in the immediate postoperative period (POD1). This mechanistic link between ultrafine tube placement, reduced intraoperative trauma and lower early drainage volume aligns with the minimally invasive tenets of U-VATS and is consistent with previous evidence that fine-bore chest tubes mitigate procedural-related tissue injury compared with large-bore alternatives (9, 15). Second, lower early drainage volume decreases the frequency of drainage bottle replacement and nursing interventions, reducing the risk of iatrogenic contamination and simplifying postoperative thoracic care, which is particularly valuable for clinical practice with limited nursing resources. Third, the reduction in early exudation and drainage is accompanied by faster resolution of pleural irritation, which further alleviates postoperative chest pain and dyspnea, creating a positive feedback loop for early rehabilitation. Importantly, the lack of difference in total drainage volume confirms that the double 8F ultrafine chest tubes achieve adequate drainage efficiency in the long term, and the reduction in early drainage volume is not due to insufficient drainage but to reduced pathological pleural exudation—eliminating clinical concerns about inadequate drainage with ultrafine tubes.

Individually, the improvements in pain control and early drainage volume may appear incremental, but their synergistic effect yields meaningful clinical benefits for U-VATS patients: the combination of reduced pain and lower early drainage volume directly reduces the incidence of clinically important complications (atelectasis and intrathoracic hemorrhage) in the double 8F group, which are associated with prolonged hospital stay, increased reintervention rate, and worse postoperative outcomes. Moreover, these improvements are achieved without increasing hospital stay, total drainage volume, or the risk of infection/air leakage, confirming that the double 8F strategy is a safe and effective drainage approach that aligns with ERAS principles. For clinical practice, these findings support the use of double 8F ultrafine chest tubes as a standardized drainage strategy for U-VATS, as it not only improves perioperative outcomes but also enhances the cost-effectiveness of postoperative care (e.g., reduced analgesic use, simplified nursing).

Nearly all prior studies were single-center (7, 9, 13–15), lacking generalizability. Our multicenter design offers key novel evidence: real-world applicability reflecting clinical practice variability, validated double 8F efficacy in heterogeneous populations (filling multicenter evidence gaps), and robust, generalizable drainage duration predictors (via PSM and linear regression). We also provide the first real-world multicenter data supporting double 8F use, complementing Kent et al. (12) multicenter expert consensus (lacking clinical outcomes). Furthermore, lead investigators at each center standardized data collection protocols before initiating data gathering. This approach mitigates data biases arising from inter-center discrepancies in the understanding of indicator definitions and measurement methods, as well as subjectively selective recording biases driven by research objectives—thereby ensuring data consistency and accuracy.

Notably, the 2024 STS expert consensus emphasizes that single large-bore tubes (19–24F) are suitable for standard lobectomy, with larger-caliber tubes recommended for cases at high risk of postoperative hemorrhage or extensive pleural adhesiolysis (12). This aligns with our observation that the single 24F group had the shortest drainage duration, reflecting its advantage in efficient drainage for uncomplicated cases. However, our multicenter cohort includes a substantial proportion of segmentectomy patients (55.04% in the double 8F group) and patients with impaired lung function, for whom the double 8F strategy may be more favorable—its ultrafine caliber minimizes intercostal nerve compression and diaphragm irritation, reducing postoperative pain and facilitating early pulmonary rehabilitation, which addresses the unmet need for personalized drainage strategies not fully covered by the consensus. Additionally, the STS consensus acknowledges surgeon preference and cost as factors in tube selection, and our study supports double 8F tubes as an evidence-based alternative that balances efficacy, safety, and patient-centered outcomes.

However, this study is a retrospective multicenter cohort study, and the choice of drainage tubes was determined by the subjective clinical preferences of individual attending surgeons at each participating center (with specific surgeons consistently using a single drainage strategy) rather than a standardized institutional protocol, which introduces potential selection bias. Although we have minimized this bias by performing 1:1:1 PSM with the inclusion of type of lung resection and other key baseline/intraoperative covariates as confounders, the non-randomized allocation of drainage strategies cannot be completely ruled out as a confounding factor for the study results. A major and important limitation of this study is the lack of a direct comparison with single 8F ultrafine chest tube drainage, a strategy that has been validated by previous studies (15) to provide adequate postoperative thoracic drainage and significant pain relief in U-VATS patients. Song et al. (15) confirmed that single 8F tubes achieve safe and effective drainage with comparable clinical outcomes to traditional large-bore tubes, and this well-supported finding highlights the clinical relevance of omitting this comparison in our research: we were unable to clarify whether the double 8F strategy offers additional benefits (e.g., more comprehensive drainage, lower complication rates) over the single 8F strategy, or whether the single 8F strategy is a more simplified and equally effective alternative for U-VATS drainage. This limitation is inherent to the retrospective observational design of our study, as single 8F tube drainage was not a clinical practice adopted by the participating centers during the study period, and thus no relevant patient data were available for analysis. Additionally, despite the multicenter design, while surgeries for enrolled patients at each center were performed by the same lead surgeon within that center, the specific impact of variations in surgical techniques and outcomes across different surgeons on postoperative drainage remains unclear. Furthermore, the drainage strategy was not associated with specific types of lung resection in this study, which reduces the potential confounding of surgical procedure on the comparison of drainage efficacy, but also limits the exploration of personalized drainage strategies for different U-VATS procedures. Another potential limitation of this study is the technical variation in chest tube placement. In the double 8F group, both ultrafine tubes were inserted through additional incisions across multiple intercostal spaces. In the 22F + 8F group, the large-bore chest tube was placed through the main surgical incision, while the fine-bore tube was inserted via an additional incision. By contrast, the 24F drain was placed exclusively through a single main surgical incision. Such differences in the number of incisions and intercostal spaces accessed may affect postoperative pain and recovery, introducing potential bias across groups. Therefore, based on the key limitations identified, future prospective randomized controlled trials (RCTs) are warranted to directly compare the clinical efficacy and safety of double 8F and single 8F ultrafine chest tube drainage in U-VATS patients; such studies will help clarify the optimal number of ultrafine chest tubes for different U-VATS surgical types (e.g., wedge resection, segmentectomy, lobectomy) and provide more refined clinical recommendations for thoracic drainage strategy selection.

In summary, our multicenter retrospective study demonstrates that several factors, including chest tube characteristics (size and quantity), surgical approach, pleural adhesions, and postoperative conditions (drainage volume, infection, air leakage, intrathoracic hemorrhage), are associated with drainage duration and while double 8F chest tubes do not reduce postoperative drainage duration, they confer clinically meaningful benefits: alleviating postoperative pain (particularly on the first postoperative day), decreasing drainage volume in the early postoperative period, and significantly lowering the incidence of specific postoperative complications. These findings highlight substantial clinical application potential for double 8F chest tubes, supporting their promising prospects for broader adoption in clinical practice.

## Data Availability

The raw data supporting the conclusions of this article will be made available by the authors, without undue reservation.
