# Supplementary material for: Postoperative drainage with double 8F ultrafine chest tubes improves pain control and reduces specific complications in uniportal thoracoscopic lung tumor resection: a retrospective multicenter cohort study
Source: Front Med (Lausanne). 2026 Apr 24;13:1806379. doi: 10.3389/fmed.2026.1806379 (PMC13154386; doi:10.3389/fmed.2026.1806379)
Supplement: Supplementary file 1 [file Table_1.DOCX]

| Variables | *P*  (overall) | *P*  (Double 8F vs 22F+8F) | *P*  (22F+8Fvs 24F) | *P*  (Double 8Fvs 24F) |
| --- | --- | --- | --- | --- |
|  |  |  |  |  |
| Hospital stay | 0.1800 | - | - | - |
| Drainage duration | **<0.0001** | >0.9999 | **<0.0001** | **<0.0001** |
| Drainage volume |  |  |  |  |
| POD1 | **<0.0001** | **<0.0001** | **<0.0001** | 0.1317 |
| POD2 | 0.1033 | - | - | - |
| POD3 | **<0.0001** | 0.4739 | **<0.0001** | **0.0032** |
| Total | 0.8690 | - | - | - |
| NRS pain score |  |  |  |  |
| POD1 | **<0.0001** | **<0.0001** | **<0.0001** | **0.0169** |
| POD2 | **<0.0001** | **<0.0001** | **<0.0001** | **0.0008** |
| POD3 | **<0.0001** | 0.0694 | 0.0748 | **<0.0001** |

Table S1. Overall difference and intergroup differences of some indicators (after propensity score matching). Kruskal-Wallis H test (p<0.05) followed by Dunn test (with Bonferroni-correction), with corrected p<0.05 indicating significance. POD, postoperative day; NRS, numerical rating scale. No statistically significant overall differences were observed for some indicators using the Kruskal‑Wallis H test, so pairwise post hoc comparisons were not conducted.

| Variables | *P*  (overall) | *P*  (Double 8F vs 22F+8F) | *P*  (22F+8Fvs 24F) | *P*  (Double 8Fvs 24F) |
| --- | --- | --- | --- | --- |
|  |  |  |  |  |
| Complication |  |  |  |  |
| Pleural effusion | **0.0005** | **0.0036** | **0.0018** | >0.9999 |
| Infection | 0.0524 | - | - | - |
| Air leakage | 0.1130 | - | - | - |
| Atelectasis | **0.0111** | 0.5070 | 0.7083 | **0.0159** |
| Post-extubation  pneumothorax | **0.0013** | 0.7377 | **0.0045** | 0.243 |
| Reintubation | >0.9999 | - | - | - |
| Intrathoracic  hemorrhage | **0.0008** | >0.9999 | 0.0549 | **0.0096** |
| Chylothorax | >0.9999 | - | - | - |

Table S2. Overall difference and intergroup differences of complications (after propensity score matching). Fisher's exact test (p<0.05) followed by Bonferroni-corrected, with corrected p<0.05 indicating significance. No statistically significant overall differences were observed for some indicators using Fisher's exact test, so pairwise post hoc comparisons were not conducted.


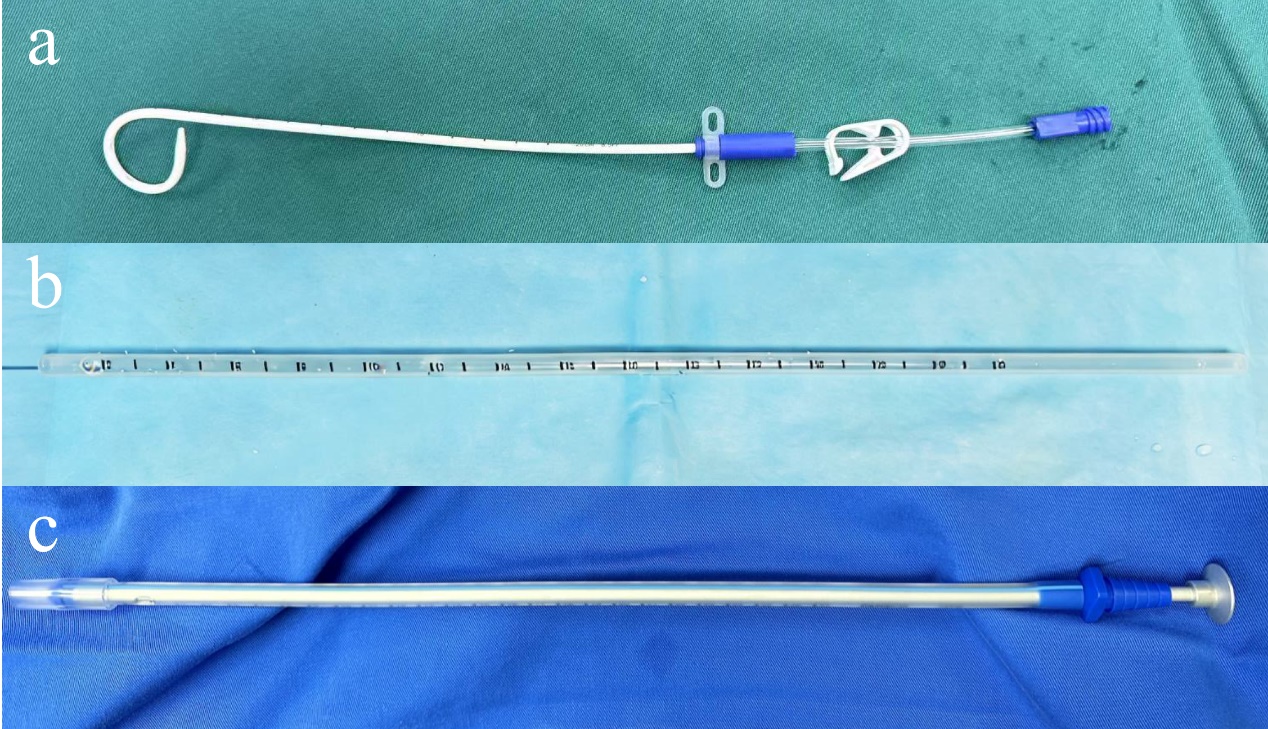


Figure S1. (a) 8F ultrafine chest tube (ABLE®; Baihe, Guangdong, China). (b) 22F chest tube (MKL, Suzhou, China). (c) 24F chest tube (Pahsco, Taiwan, China).


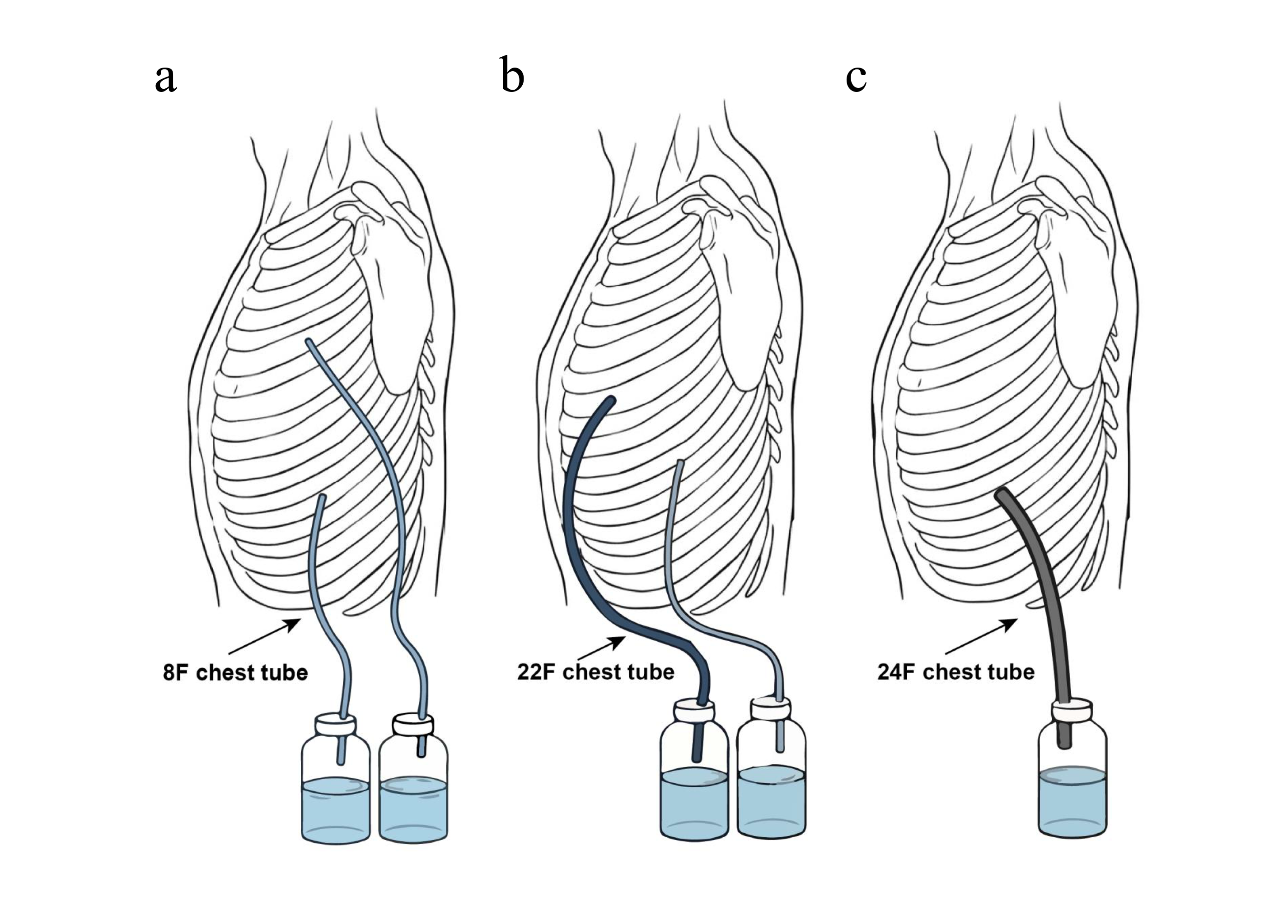


Figure S2: All tubes, whether fine‑bore or large‑bore, were connected to a water‑sealed drainage system. (a) In the double 8F group, two 8F chest tubes were placed postoperatively: one inferior tube in the 7th–8th intercostal space at the midaxillary line, and one superior tube in the 3rd intercostal space at the midaxillary line. (b) In the 22F+8F group, an 8F chest tube was positioned in the 7th–8th intercostal space at the midaxillary line, and a 22F chest tube in the 4th–5th intercostal space at the anterior axillary line. (c) In the 24F group, a single 24F chest tube was inserted via the 7th intercostal observation port.

Figure S3. Love plot for propensity score matching balance diagnostics


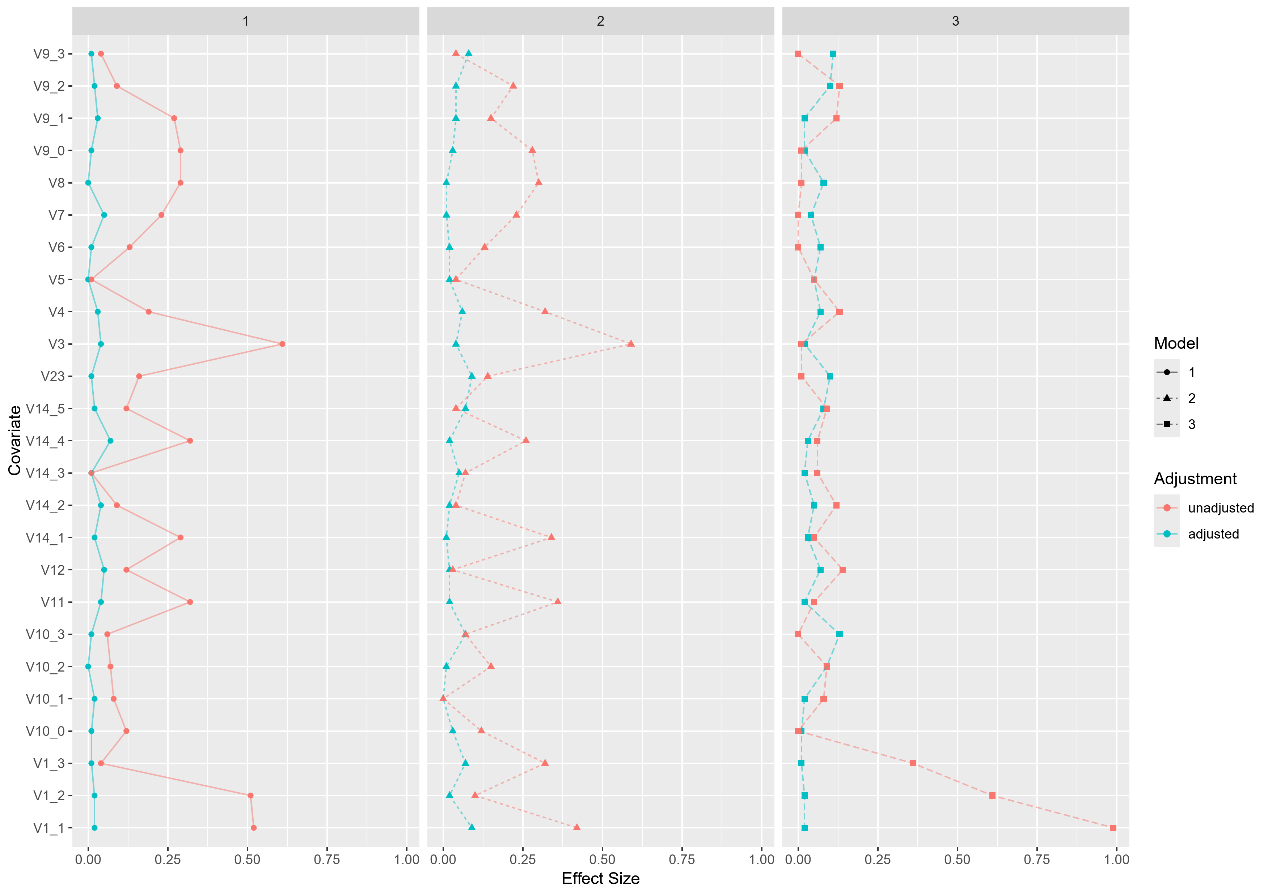


The Love plot shows the standardized mean differences (SMDs) of all baseline and intraoperative covariates included in the propensity score model before (red dots) and after (blue dots) 1:1:1 PSM. The vertical dashed line represents the SMD threshold of 0.1; all covariates fall within the range of SMD < 0.1 after matching, indicating adequate intergroup balance.

Figure S4. Covariate balance in the PSM-matched cohort


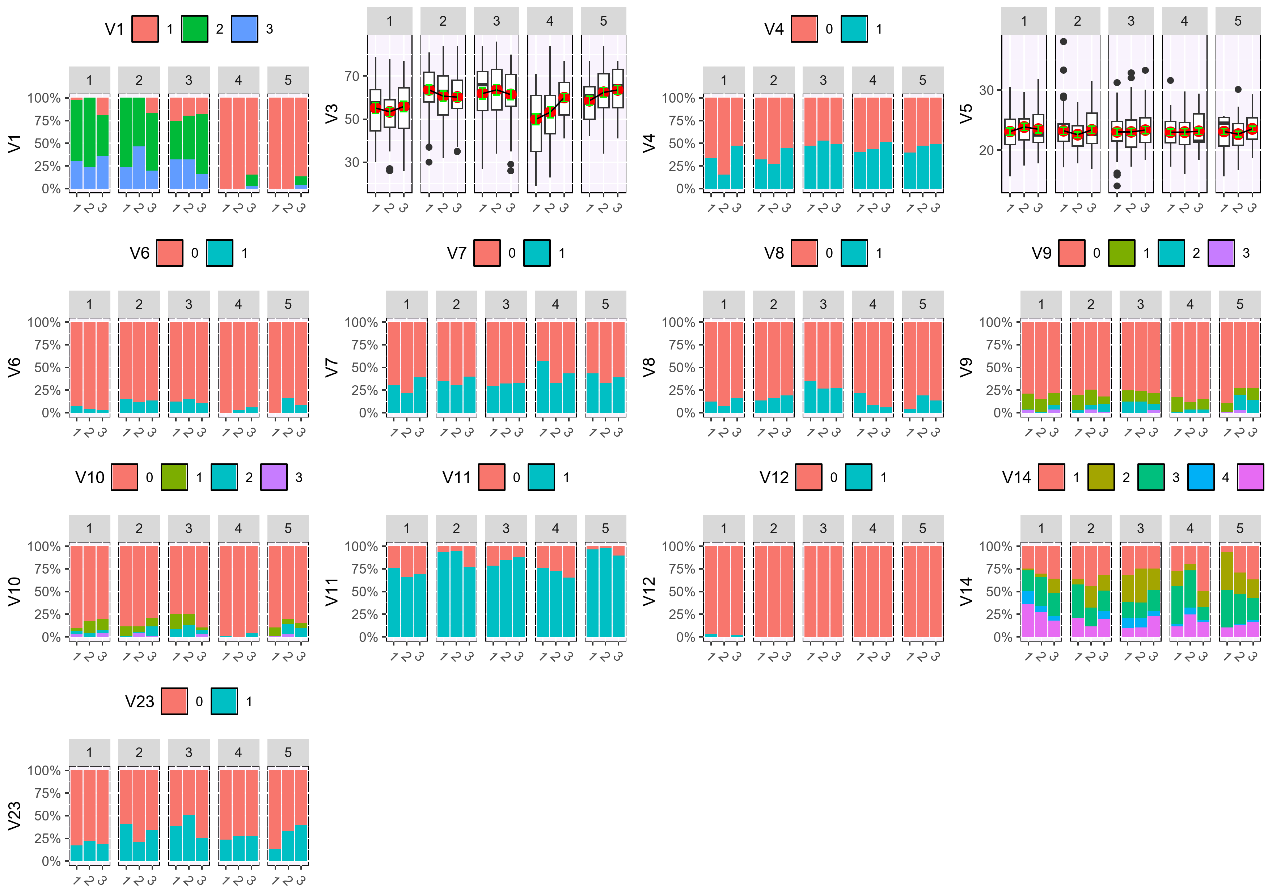


Distribution of baseline and intraoperative covariates (V1-V23, Table 1) across the double 8F, 22F+8F and single 24F groups after 1:1:1 propensity score matching. Percentile distribution (0%, 25%, 50%, 75%, 100%) and numerical consistency of all covariates confirm satisfactory intergroup balance (SMD < 0.1), validating the comparability of the three matched cohorts.
